# Measuring recent cannabis use across modes of delivery: Development and validation of the Cannabis Engagement Assessment

**DOI:** 10.1016/j.abrep.2022.100413

**Published:** 2022-02-08

**Authors:** Magdalen G. Schluter, David C. Hodgins

**Affiliations:** Department of Psychology, University of Calgary, Calgary, AB T2N 1N4, Canada

**Keywords:** Cannabis, Cannabis Engagement Assessment (CEA), Scale development, Instrument validation, Test-retest reliability

## Abstract

**Introduction:**

Methods of cannabis engagement have proliferated in recent years, which many self-report measures do not adequately capture. There is a clear need for self-report measures that capture current patterns of cannabis use across a range of methods, and that can be used to track changes over time. The current study developed the Cannabis Engagement Assessment (CEA), a self-report measure of past month cannabis use across dry flower, concentrate, and edible products.

**Methods:**

A sample of 349 participants from the undergraduate student population and broader community were recruited. To examine convergent validity of the CEA, participants completed measures of cannabis engagement, cannabis misuse, and use-related problems. To assess divergent validity, participants also completed measures of depression and alcohol use problems. Criterion and test–retest reliability were examined in a subset of 65 participants who re-completed the CEA and a timeline follow-back interview (TLFB).

**Results:**

Indicators of cannabis use frequency and quantity showed good convergence with measures of cannabis use patterns, problematic engagement, and cannabis use-related problems. Divergent validity of the CEA was supported by lower associations with alcohol use problems and depression symptoms. The CEA also showed good test–retest reliability and convergence with estimates of frequency and quantity of cannabis use from the TLFB.

**Conclusions:**

The CEA is a viable self-report measure of cannabis use that is representative of current patterns of recreational cannabis engagement. Its focus on cannabis use in the preceding 30 days also lends itself to measuring changes in use over time.

## Highlights

• The CEA is consistent with expert-recommended guidelines for quantifying cannabis use.
• The CEA is representative of current patterns of recreational cannabis engagement.
• Estimates of cannabis use show strong associations with the timeline followback method.
• The CEA shows good test–retest reliability over a one-week period.

## 1 Introduction

Cannabis is the most used illicit substance around the globe. Worldwide, the annual prevalence is approximately 3.9% (United Nations Office on Drugs and Crime, 2020), compared to only a 1.2% annual prevalence of opioid use. In Canada, 14.8% of individuals 15-years and older reported using cannabis in 2017 just prior to legalization of recreational use (Health Canada, 2020). With the legalization of cannabis in North America and increased interest in surveillance, questions have been raised of how to define a standard unit, developing low-risk guidelines, and understanding the relationships between cannabis-related harms and the frequency and quantity of use (Zeisser et al., 2012, Asbridge et al., 2014, Tomko et al., 2019, Hammond et al., 2020). Until recently, the field also lacked expert consensus on minimum standards for quantifying cannabis use. The iCannToolkit (Lorenzetti et al., 2021), which represents a new hierarchical framework for quantifying cannabis use, also indicates a critical need to develop self-report assessments consistent with this framework. The issue of accurate self-report assessment is complicated by the proliferation of ways in which individuals now use cannabis, which many existing self-report measures do not capture adequately (e.g., MSHQ; Bonn-Miller & Zvolensky, 2009). Studying the effectiveness of interventions for problematic cannabis use also requires the ability to track changes in cannabis use over time, which is another common limitation of some measures (e.g., DFAQ-CU; Cuttler & Spradlin, 2017). Given these issues, we sought to develop a reliable and valid self-report measure that captures the diversity of ways in which people engage with cannabis and can be used to track changes in cannabis use over time.

Smoking has historically been the primary mode of cannabis use (Gunn, Aston, Sokolovsky, White, & Jackson, 2020) though its popularity has decreased (Health Canada, 2020). Legalization and other trends are increasing the availability and use of alternative modes of cannabis such as edibles and vaping (Borodovsky et al., 2016, Schauer et al., 2016). Vaping and dabbing are other common ways in which cannabis can be inhaled. Vaping refers to the inhalation of cannabis vapors that are created by heating a cannabis product without burning. Dabbing refers to inhaling the smoke from concentrated cannabis products which are heated on a hot surface. Vape pens have also become increasingly popular, and cannabis companies have begun partnering with beverage companies to produce cannabis-infused sodas. Other methods include ingestion through use of concentrates or edibles, and through skin absorption.

Modes of cannabis consumption differ in their Δ9-tetrahydrocannabinol (THC) potency and timing of delivery (Loflin & Earleywine, 2014), bioavailability and peak plasma concentration (Sharma, Murthy, & Bharath, 2012), subjective effects (Okey & Meier, 2020), and behavioural reinforcement (O'Brien, 2001). These factors may have important implications for understanding the relationships between frequency and quantity of use and cannabis-related harm, and with patterns of use over time. For example, ‘dabbing’ may be more likely than dry flower products to contribute to symptoms of tolerance and withdrawal (Loflin & Earleywine, 2014). As such, measures of cannabis engagement should assess use across a range of methods.

The Marijuana Smoking History Questionnaire (MSHQ; Bonn-Miller & Zvolensky, 2009), the most used measure of cannabis use, assesses the use of cannabis through smoking only and fails to accurately measure frequency and quantity of consumption. As noted by Cuttler and Spradlin (2017), the MSHQ assesses frequency on an 8-point Likert scale without verbal anchors for each response option. As such, responses are likely to be highly subjective. Additionally, the highest endpoint is listed as “more than once a day” but does not further assess frequency within a single day. It is possible that individuals who use cannabis twice a day may be at lower risk for developing harms than an individual who uses cannabis four times each day.

The Daily Sessions, Frequency, Age of Onset, and Quantity of Cannabis Use Inventory (DFAQ-CU; Cuttler & Spradlin, 2017) was more recently developed with preliminary psychometric properties reported for an undergraduate sample. This measure overcomes many limitations of the MSHQ, including the assessment across multiple modes of cannabis, and is useful for assessing an individual’s general pattern of cannabis use. However, it is not applicable for intervention research where cannabis engagement must be measured within a specified reporting window and tracked reliably over time. The DFAQ-CU does ask participants to estimate how many days in the past week and month they used cannabis. However, it does not provide a detailed assessment of quantity within those windows. To our knowledge, the Timeline Follow-back Method (TLFB; Sobell et al., 1996) is currently the only measure that does this. However, this method can be time-consuming, as it requires the participant to retrospectively complete a detailed record of their use in a window of time (e.g., one month, three months, 12 months) using prompts provided by an interviewer. Self-report versions of the TLFB have been developed (Pedersen et al., 2012), though they are also time intensive. A self-report measure that can be completed more quickly may be more desirable when a participant is completing a host of other measures as well.

In sum, measurement of cannabis use has generally not kept pace with shifts in patterns and modes of engagement and measures that do capture the diversity of ways in which cannabis is used are not designed to track changes over time. Therefore, we sought to develop the Cannabis Engagement Assessment (CEA), a self-report measure of use in the preceding month. To ensure content validity (Messick, 1995, Vogt et al., 2004), individuals who use cannabis recreationally were engaged in qualitative interviews to examine current patterns and modes of cannabis use, amounts, and other factors that could be relevant for assessing cannabis use. The information obtained during the qualitative interviews, in conjunction with consideration of the literature, was used to guide development of the CEA. Types of cannabis products are divided into three major categories, consistent with how participants described the types of cannabis products: dried cannabis flower products, cannabis concentrates, and edible products. Visual depictions of both dried products and various concentrates, adapted with permission from Goodman, Leos-Toro, and Hammond (2019), are also included to help participants estimate amounts used.

The current study aimed to examine the psychometric properties of the CEA. As the measure is intended to be able to assess changes in cannabis use over time, we also aimed to determine whether information from the CEA could be used to provide an estimate of overall cannabis and THC used in the preceding 30 days.

## 2 Materials and methods

### 2.1 Participants and procedure

The sample consisted of 349 participants drawn from the community (*n* = 144) and the undergraduate student population (*n* = 205). Inclusion criteria were: (a) 18-years or older, (b) cannabis use within the past month, and (c) use of cannabis for recreational (i.e., not solely medical) purposes. Community participants were recruited through online media advertisements on Kijiji, a popular classifieds website, and Facebook. Undergraduate students were recruited through the university’s online Research Participation System, which allows undergraduate psychology students to participate in ongoing research studies for partial course credit.

In part 1 of the study, participants completed a series of self-report questionnaires, including the CEA on Qualtrics. Participants were asked if they would be interested in part 2, a follow-up interview approximately one-week later. Participants in part 2 (15 community and 50 students) re-completed the CEA and participated in a timeline follow-back interview over Microsoft Teams. Community participants were provided a $5 e-gift card for their participation in part 1 and an additional CAD $15 on the e-gift card for part 2. Undergraduate participants received 0.5 credits for participation in the survey, and another 0.5 credits if they also participated in the follow-up interview, consistent with university standards.

### 2.2 Measures

#### 2.2.1 Demographic questionnaire

A lab-developed questionnaire recorded age, gender, marital status, level of education, and household income.

#### 2.2.2 Cannabis Engagement Assessment (CEA)

The CEA (Appendix A) contains 30 questions that assess the quantity, frequency of use, and method of consumption for dried cannabis products (excluding edibles), cannabis concentrates, and edible products in the previous 30 days. Two additional sections assess other factors associated with cannabis use and history of use.

#### 2.2.3 Daily Sessions, Frequency, Age of Onset, and Quantity of Cannabis Use Inventory (DFAQ-CU; Cuttler & Spradlin, 2017)

The DFAQ-CU (Cuttler & Spradlin, 2017) is a self-report inventory of cannabis use that assesses a general pattern of frequency and quantity of cannabis use, and age of onset. Standardized mean scores are calculated for each of the 6 factors: daily sessions, frequency, dry product quantity, concentrate quantity, edible quantity, and age of onset. The internal reliability for the current study ranged from α = 0.69 (daily sessions) to α = 0.85 (frequency).

#### 2.2.4 Cannabis Use Disorders Identification Test – Revised (CUDIT-R; Adamson et al., 2010)

The CUDIT-R is an eight-item measure of problematic cannabis use in the past 6 months (Adamson et al., 2010). The total score internal reliability for the current study was α = 0.83.

#### 2.2.5 Marijuana Problem Scale (MPS; Stephens et al., 1994, Stephens et al., 2000)

The MPS is a 19-item measure of problems associated with cannabis use in the previous month. Questions assess the impact of cannabis use across social, financial, work, physical health, cognition, self-esteem, motivation, and legal domains. Reliability of the total score for the current study was α = 0.95.

#### 2.2.6 Alcohol Use Disorders Identification Test – Consumption (AUDIT-C; Bush, Kivlahan, McDonell, Fihn, & Bradley, 1998)

The AUDIT-C is a three-item measure of problematic alcohol use that is used to identify individuals who exhibit high risk drinking behaviours or likely alcohol use disorders. Reliability in the current study was α = 0.73.

#### 2.2.7 Patient Health Questionnaire (PHQ-9; Kroenke et al., 2001, Kroenke and Spitzer, 2002)

The PHQ-9 is a self-report screening measure of depression symptoms. It has excellent internal reliability (a = 0.86–0.89) and criterion validity (Kroenke et al., 2001). Reliability for the current study was α = 0.90.

#### 2.2.8 Timeline Follow-Back Method (TLFB; Sobell et al., 1996)

The TLFB for marijuana is an interview-based assessment of substance use that asks individuals to estimate their marijuana use over a specified interval using a calendar format. With cannabis use, the TLFB shows good test–retest reliability (Robinson, Sobell, Sobell, & Leo, 2014) and generally high construct validity (Hjorthøj, Hjorthøj, & Nordentoft, 2012). For this study, participants were asked to fill out the calendar with their cannabis use in the preceding 30 days. The standard prompts and instructions were used to guide participants. Of note, the TLFB for marijuana asks participants to record only how many joints, and the “average” size of the joints used for each day that they used cannabis. Thus, we also asked participants, consistent with the updated TLFB used by Martin-Willett et al. (2020), to distinguish between flower, concentrate, and edible use on the TLFB. Participants were also encouraged to estimate the THC content of the cannabis that they reported on the TLFB. The following variables were calculated from the information obtained on the TLFB: number of days of cannabis use, number of days of use for each mode of engagement, average THC content for each type of product (if known), the overall amount used for each type of product, and average amount used per day for each mode of engagement.

### 2.3 Data analysis

To evaluate the psychometric properties, we examined convergent and divergent validity, and test–retest reliability. Responses to CEA items were used to estimate overall amounts of THC used in the preceding month for each mode of engagement (dried flower, concentrates, and edibles). The total amount of cannabis was calculated for dry flower products, but not for concentrates or edibles. THC used for concentrates was calculated by multiplying the reported THC percentage by the reported mg of concentrate used. One ‘puff’ of concentrate was assumed to contain 5.2 mg of concentrate (Varlet et al., 2016). Bivariate Kendall’s tau correlation coefficients were calculated between measures of frequency, quantity, history of use, and external measures. We predicted that we would see higher associations between the CUDIT-R and CEA, and between the MPS and CUDIT-R, than between the CEA and the AUDIT-C and PHQ-9.

For continuous variables, test–retest reliability was examined using the intraclass correlation coefficient (ICC). Values between 0.5 and 0.75 suggest moderate reliability, values between 0.75 and 0.90 suggest good reliability, and values above 0.90 suggest excellent reliability (Koo & Li, 2016). Kappa coefficients and McNemar’s test were used to examine test–retest reliability of categorical variables. Kappa values closer to +1 and p > 0.05 on the McNemar test indicate higher consistency between test and retest responses.

Finally, convergent validity of the CEA for assessing frequency and quantity of cannabis use across the three modes of engagement was examined by comparing CEA and TLFB variables from part 2 using correlation coefficients, the mean difference between the two scores, and the 95% confidence interval of the mean difference.

## 3 Results

### 3.1 Descriptive statistics

The community sample consisted of 144 participants (104 male, 72.2%), aged 18 to 63 years (M = 31.68, *SD* = 7.85), mostly Caucasian (*n* = 96, 66.7%), legally married (*n* = 72, 50.0%), and employed full-time (*n* = 92, 63.9%). The student sample consisted of 205 participants (26 male, 12.7%), aged 18–36 years (M = 20.20, *SD* = 3.06), mostly Caucasian (*n* = 123, 60.0%), and unmarried (*n* = 187, 91.2%).

Cannabis engagement characteristics are reported in Table 1. Participants reported using cannabis a mean of 12.64 days in the past month (*SD* = 9.05) among the community sample and 7.19 days (*SD* = 8.26) in the student sample. Overall, the community sample reported heavier use and greater related problems than the student sample; 18% had a CUDIT-R in the hazardous range (8–11; Adamson et al., 2010) and 64% fell in the range of a possible cannabis use disorder (≥12). In contrast, only 9.9% and 18% of the student sample fell in these ranges, respectively. Dried cannabis products were the most used mode (*n* = 298), followed by edibles (*n* = 201) and concentrates (*n* = 178). Among individuals who used concentrates, only 72 (40.5%) provided an estimate of quantity used in mg or puffs.

**Table 1:** Cannabis engagement characteristics and scores on external measures across samples.

	Total Sample (N = 349)				Community (N = 144)				Student (N = 205)			
	n	%	M	SD	n	%	M	SD	n	%	M	SD
Use – Dried Cannabis Product	298	85.39			132	92.67			166	80.98		
Frequency (days)			7.31	8.35			11.46	8.66			5.89	7.37
Daily sessions			2.10	1.94			2.31	1.21			1.93	2.36
Total cannabis used (g)			26.93	60.37			46.73	78.17			10.90	33.07
THC content (%)			20.14	16.70			16.43	11.44			26.37	21.74
THC total (g)			9.71	18.37			11.02	19.25			7.51	16.56

Use – Concentrated Cannabis Products	178	51.00			84	58.33			94	45.85		
Frequency (days)			6.88	7.48			7.75	6.81			6.10	7.98
Daily sessions			2.19	2.01			2.63	1.25			1.90	2.64
THC content (%)			34.08	31.62			22.60	24.96			49.40	33.32
THC total (g)^a^			1.66	7.04			2.70	10.28			0.76	1.41

Use – Edible Products	201	57.59			105	72.92			96	46.83		
Frequency (days)			6.53	7.01			9.96	7.77			2.78	3.19
Daily sessions			2.09	1.72			2.13	1.31			2.05	2.09
THC total (g)			0.53	2.23			0.71	2.75			0.20	0.46

Frequency of overall cannabis use (days)			9.36	8.98			12.64	9.05			7.19	8.26
Age of First Use			18.84	4.60			20.96	5.94			17.33	2.40
Age of Regular Use			21.20	5.46			24.77	7.28			19.27	2.62
CUDIT-R			9.78	6.35			13.78	5.57			6.94	5.24
MPS			27.79	9.08			15.63	9.26			4.01	4.95
PHQ-9				8.82	6.39		10.92	6.32			7.35	6.03
AUDIT-C				3.95	2.37		4.71	2.47			3.42	2.14

a Calculated from estimate of 5.2 mg per puff of concentrate.

### 3.2 Associations with external measures

In support of convergent validity, indicators of cannabis use quantity, frequency, and age of onset on the CEA showed moderate to high positive correlations with similar DFAQ-CU subscales. As shown in Table 2, sessions of cannabis use across dry and concentrate products correlated moderately with the DFAQ-CU daily sessions factor (*τ*’s = 0.30–0.40). Sessions of edible use showed only a weak association. This may be, in part, because the CEA provides an operational definition of a “session*”* whereas the CFAQ-CU does not. In qualitative interviews, participants noted potential differences across participants on the definition of an “occasion” on the MSHQ. We anticipated a similar issue with the term “session” and included a definition in the CEA to enhance content validity. Similarly, except for edibles, all indicators of cannabis frequency in the past month showed moderate positive associations with the frequency factor on the DFAQ-CU (*τ*’s = 0.30–0.63). Estimates of the quantity of cannabis and THC used in the previous month also showed small to moderate associations with the quantity factors on the DFAQ-CU. The weakest association was observed between THC used through concentrates and the concentrate quantity factor (*τ* = 0.18) and the strongest association was between the amount of dry product used and the marijuana quantity factor (*τ* = 0.47). The low concentrate association is unsurprising, as the questions on the DFAQ-CU comprising this factor refer to the quantity of hits, whereas the CEA estimates the quantity of THC consumed. As such, the CEA and DFAQ-CU are assessing different variables. Finally, strong association were observed between age of onset questions on the CEA and the age of onset factor on the DFAQ-CU (*τ*’s = 0.75 and 0.81).

**Table 2:** Kendall’s Tau correlation coefficients.

		DFAQ-CU									
CEA		Daily Sessions	Frequency	Marijuana Quantity	Concentrate Quantity	Edible Quantity	Age of Onset	CUDIT-R	MPS	PHQ-9	AUDIT-C
Daily Sessions	Dry	0.39**						0.38**	0.24**	0.15**	0.29**
	Concentrate	0.40**						0.41**	0.35**	0.27**	0.25**
	Edibles	0.06						0.26**	0.21**	0.17**	0.28**
Frequency	Days Used – Dry		0.55**					0.44**	0.26**	0.10**	0.14**
	Days Used – Concentrate		0.30**					0.26**	0.13**	0.13**	0.13**
	Days Used – Edibles		0.23**					0.27**	0.24**	0.18**	0.19**
	Overall Days Used		0.63**					0.44**	0.22**	0.12**	0.13**
Dry product – Quantity	Total Cannabis			0.47**				0.50**	0.33**	0.18**	0.21**
	Total THC			0.41**				0.27**	0.23*	0.15*	0.12*
Concentrate Quantity	Total THC				0.18*			0.1	−0.23*	−0.18	−0.11
Edible Quantity	Cannabis					0.25		−0.11	−0.07	−0.1	−0.05
	Total THC					0.35**		0.23**	0.08	0.11	0.16*
History	Age first use						0.81**	0.06	0.21**	0.15**	−0.03
	Age regular use						0.75**	0.06	0.17**	0.13**	<0.01

* p < .05.

** p < .01.

The CUDIT-R total score generally showed moderate positive correlations with indicators of cannabis use frequency, including the number of daily sessions (*τ*’s = 0.26–0.44; Table 2). Associations between cannabis use frequency and quantity and the AUDIT-C were smaller than those seen with the CUDIT-R (*τ*’s = 0.13–0.29). A similar pattern was observed with the associations between the CEA and the MPS (|*τ*|’s = 0.07–0.35). The PHQ-9 also showed weak to small associations with the CEA (|*τ*|’s = 0.101–0.27). Finally, weaker associations were also observed between measures of cannabis use frequency and quantity and the MPS (|*τ*|’s = 0.05–0.21) than with the CUDIT-R.

### 3.3 Criterion validity

For participants who completed part 2, correlations between estimates of cannabis frequency and quantity obtained by the CEA and timeline follow-back interviews (TLFB) ranged from 0.65 to 1.00 (Table 3), indicating strong to very strong associations. On average, participants reported 1.36 more greater number of days of dry product use on the CEA compared to the TLFB (*p* = 0.05). The total amount of cannabis and THC reported on the CEA were also higher (*p* = 0.004 and 0.03). Regarding concentrated cannabis products and edibles, the average reported days of use and THC content did not differ significantly (*p*s > 0.21). Finally, participants reported a similar number of separate days of cannabis use on both the CEA and TLFB (*p* = 0.58).

**Table 3:** Comparison of the CEA and Timeline Follow-Back.

		*r*	Difference between means			
			M	*SD*	95% CI	*p*
	Dry Product					
	Days Used	0.89**	1.36	4.63	0.003, 2.72	0.05
	THC content	0.78**	−1.22	3.74	−2.92, 0.48	0.15
	Total cannabis	0.87**	5.21	11.09	1.80, 8.63	0.004

Concentrate						
	Days Used	0.80**	−0.82	5.60	−2.80, 1.17	0.41
	THC content	0.84**	2.37	14.28	−6.26, 10.99	0.56

Edibles						
	Days Used	0.84**	0.48	1.78	−0.29, 1.25	0.21
	Total cannabis (grams)^a^	1.00*	41.43	64.59	−119.02, 109.81	0.38
	Total THC (mg)	0.65**^b^	30.29	137.72	−49.23, 129.33	0.43
Separate Days		0.86**	0.38	5.52	−1.75, 0.98	0.58

*Note:* these results were calculated from participants who completed part 2 of the study (*n* = 65).

* p < .05.

** p < .01.

a Small sample size (n = 3).

b Kendall’s tau correlation coefficient

### 3.4 Test-retest reliability

Across items, ICCs generally ranged from 0.58 to 0.99 (Table 4), suggesting moderate to excellent reliability. However, questions pertaining to the average amount of concentrated product per session showed poor reliability (ICC 0.08). Regarding categorical variables, kappa values ranged from 0.73 to 1.00, suggesting that participants showed substantial to almost perfect agreement in their responses. McNemar’s tests were non-significant (*p*’s = 0.07–1.00), also supporting test–retest reliability.

**Table 4:** Reliability coefficients for CEA items.

	ICC	Kappa	McNemar
Dry Product			
Days of use	0.88		
Main way of use		0.86	*p* = .56
Sessions per day	0.69		
Total sessions	0.80		
Amount of cannabis per session	0.43		
THC content	0.99		
Total THC consumed	0.48		

Concentrates			
Days of use	0.83		
Sessions per day	0.08		
Total sessions	0.58		
THC content	0.98		

Edibles			
Days of use	0.67		
Sessions per day	0.61		
Total sessions (edibles)	0.58		
THC content (edibles)	0.96		

Overall Days	0.93		
Setting (alone vs other people)		0.73	*p* = .39
Source of cannabis		0.92	*p* = .50
Use with other substances		0.73	*p =* .22
Age of first use	0.98		
Age regular use	0.98		
Years of regular use	0.73		
Ever tried to cut down		0.75	*p* = .07
Ever sought treatment		1.00	*p =* 1.00

*Note:* these results were calculated from participants who completed part 2 of the study (*n* = 65).

## 4 Discussion

The project aimed to assess the psychometric properties of the Cannabis Engagement Assessment (CEA), a self-report measure of recent cannabis engagement (i.e., in the past 30 days). Qualitative interviews with individuals who use cannabis recreationally, in conjunction with the existing literature, guided the development of items on the CEA reflecting current use modes and patterns. Overall, the CEA showed good psychometric properties, supporting its utility of a measure of recreational cannabis use that can be used to track changes over time.

The CEA includes items that screen for lifetime cannabis use and overall frequency of cannabis use in the preceding 30 days. These questions are consistent with the recently established guidelines for quantifying self-reported cannabis use (Lorenzetti et al., 2021). The iCann toolkit also recommends that all self-report questionnaires include an item that assesses when the participant most recently used cannabis. Considering this recommendation, we have also added such a question to the CEA such that the CEA meets the expert-recommended universal guidelines for quantifying self-reported cannabis use.

While the importance of assessing both frequency and quantity of cannabis is recognized (Volkow et al., 2016), there is significant heterogeneity in the literature regarding the best operationalization of cannabis reduction (Lee et al., 2019). Given these ongoing issues, the CEA includes several indices of cannabis engagement that integrate both frequency and quantity (e.g., the overall amount of THC consumed through a given mode). Total cannabis and THC used in the past month were able to be calculated for each type of cannabis. For concentrated cannabis products, approximately 60% of participants could not estimate the concentrate amount in mg or puffs/hits per session. For the participants who indicated the amount they used in puffs, we used a standard estimate of 5.2 mg/puff from Varlet et al. (2016) to calculate the amount of mg. of cannabis product and subsequent THC estimates based on the THC concentration. While this is a rough estimate only, it is a first step in estimating overall amounts of THC consumed through recreational cannabis use, considering both quantity and frequency. It is important to note that other factors such as bioavailability may impact cannabis use and are not able to be estimated in a brief self-report measure.

The CEA showed generally strong psychometric properties. Estimates of quantity and frequency obtained by the CEA showed strong associations with the same estimates obtained through timeline follow-back interviews, supporting its criterion validity. Except for total dry cannabis used, differences between the CEA and TLFB were non-significant. On average, individuals reported 5.21 g more of dry cannabis product on the CEA than the TLFB. This is unsurprising as one of the variables used to calculate this estimate, amount used per session, showed poor temporal stability (ICC = 0.48). Sessions of concentrate per day also showed low-test retest reliability (ICC = 0.08). While some variation in patterns of cannabis use across weeks is to be expected, it is unlikely that the low test–retest reliability for concentrate sessions was due to a significant change in pattern of use during the week between time 1 and time 2; Test-retest reliability across other questions was good, suggesting that individuals did not markedly change their pattern of cannabis use. Rather, it appears that participantsstruggled to consistently estimate how much product they typically used in a single session in the previous month. To address this, we modified the questions on the final version of the CEA to ask how much dry product, on average, was used in a single day, rather than in a single session, and the average number of ‘hits’ per day for concentrated products. As noted by Lorenzetti et al. (2021), quantification based on a more general metric is recommended in situations where the individual cannot accurately estimate the quantity of cannabis use. Estimating the number of “hits” per day also provides a metric by which to assess changes over time, which is consistent with the goals of the CEA.

Other estimates of test–retest reliability were moderate to high, supporting the temporal stability of the CEA over a one-week span. As such, the CEA may be useful in tracking changes in use over time. While interventions for cannabis misuse have typically emphasized abstinence-only outcomes, controlled use is also a popular treatment goal (Tomko et al., 2019). Evaluation of the utility of interventions for cannabis use among individuals who do not want to achieve complete abstinence required the ability to measure changes in cannabis engagement over time. Other measures, such as the DFAQ-CU, while showing good preliminary psychometric properties, do not provide detailed information of use within a specified timeframe. The CEA, in contrast, is designed to assess cannabis use in the preceding 30 days.

Comparison of the CEA to other measures also provided support for its utility. When compared to related factors on the DFAQ-CU, indicators of frequency and quantity of cannabis use on the CEA generally showed small to moderate associations. One exception is daily sessions of edible use, which showed only a weak association to the DFAQ-CU daily sessions factor. However, the daily sessions factor is only comprised of items that assess sessions of concentrate and dry product use. Therefore, it is unsurprising that sessions of edible use were unrelated to this factor. Overall, the pattern of results suggests that the CEA assesses related, but not identical, aspects of cannabis engagement as compared to the DFAQ-CU.

Estimates of frequency generally showed stronger associations with the CUDIT-R than measures of other less directly related constructs. Moderate correlations were generally observed between the CUDIT-R and indicators of cannabis use frequency and quantity. The AUDIT-C showed smaller associations with these same measures, supporting the CEA’s divergent validity. Overall, the CEA shows good psychometric properties which supports its use as a self-report measure of recreational cannabis use in the preceding 30 days.

This study has several limitations. First, the number of community participants who agreed to participate in the follow-up interview was small, despite the higher gift card value for participating in the follow-up interview compared to the online survey. Therefore, test–retest reliability, and the level of agreement between the CEA and TLFB could not be separately examined for the two samples. Second, the study relied on convenience samples. The community sample was predominantly male whereas the student sample was predominantly female. This is not surprising, given the higher prevalence of cannabis use and misuse among males than females in community samples (Calakos, Bhatt, Foster, & Cosgrove, 2017). In contrast, student samples tend to have an overrepresentation of females (Dickinson, Adelson, & Owen, 2012). Given the observed imbalance within each sample, as well as between the two, it was not possible to assess gender differences. Third, a few CEA variables showed low test–retest reliability. We have therefore eliminated or changed the questions to improve reliability. Lastly, the study did not include a biological or other objective measure of cannabis use. Future research should also include other methods for assessing cannabis use such as biological measures to further examine the psychometric properties of the CEA.

## 5 Conclusions

In sum, the CEA is a viable self-report measure of cannabis use and that is more representative of current patterns of recreational cannabis engagement than other measures. Its questions are also consistent with expert-recommended guidelines for universal screening. Estimates of frequency and quantity of cannabis showed good test–retest reliability and convergence with estimates from the TLFB. Moreover, its ability to estimate overall amounts of cannabis and THC consumed through three main types of cannabis lends itself to measuring changes in use over time. Future research may want to examine the CEA in more diverse samples and using more accurate measures of cannabis use such as daily tracking.

### CRediT authorship contribution statement

**Magdalen G. Schluter:** Conceptualization, Methodology, Investigation, Formal analysis. **David C. Hodgins:** Conceptualization, Methodology, Supervision.

## Declaration of Competing Interest

The authors declare that they have no known competing financial interests or personal relationships that could have appeared to influence the work reported in this paper.
