# Supplementary material for: Measuring recent cannabis use across modes of delivery: Development and validation of the Cannabis Engagement Assessment
Source: Addict Behav Rep. 2022 Feb 8;15:100413. doi: 10.1016/j.abrep.2022.100413 (PMC9006737; doi:10.1016/j.abrep.2022.100413)
Supplement: Supplementary data 1 [file mmc1.docx]

**Appendix A**

**Cannabis Engagement Assessment (CEA)**

The following questions ask about your cannabis use in the past month (30 days). Cannabis includes marijuana, weed, grass, bud, oil, or any other use of the cannabis plant.

**Part I – Types and Methods of Cannabis Use**

1a. Have you ever used cannabis?

YES NO *end survey*

1b. Have you used cannabis in the past month?

YES NO *end survey*

1c. When did you last use cannabis?

1. Today
2. Yesterday
3. In the past week
4. 1 to 2 weeks ago
5. 2 to 3 weeks ago
6. 3 to 4 weeks ago

Bud, weed, and other dried cannabis leaf products (**NOT including concentrates or edibles**)

1. How many **days** in the past month did you use weed, bud, or other dried cannabis leaf products (excluding concentrates and edibles)?

____ *If 0, skip to Question 3*

1. In the past month, what is the **main** **way** that you used bud, weed, or other dried cannabis leaf products (not including edibles)?
2. Pipe/one-hitter
3. Bong/hooka
4. Joint/blunt
5. Hot knives
6. Parachute
7. Dried herb vape pen
8. Other: _____
9. On the days that you used bud, weed, or other dried leaf cannabis in the past month, how many **sessions** (blocks of time separated by at least 2-hours where you did not use cannabis) did you typically have **in a single day**?
10. 1
11. 2
12. 3
13. 4
14. 5
15. More than 5 (specify number): __

Please refer to the image below depicting various quantities of marijuana. The image is not to scale; The bottle cap is included to help provide size perspective.

| 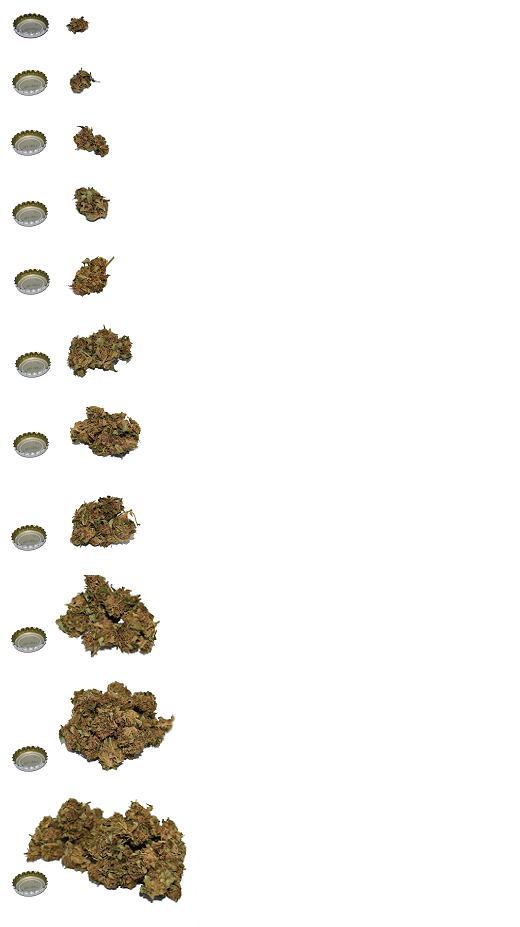 | 0.125 (one eighth) gram  0.25 (one quarter) gram  0.5 (one half) gram  0.75 (three quarters) gram  One gram | 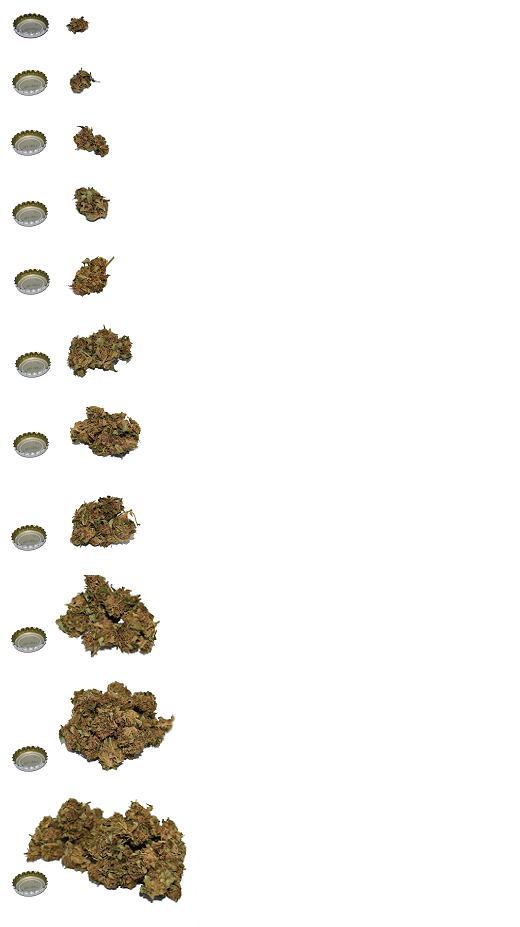 | 2 grams  3 grams  3.5 grams (eighth ounce)  7 g (quarter ounce) |
| --- | --- | --- | --- |

1. In the past month, how much bud, weed, or other dried products did you typically use **in a single day**?
2. .25g (quarter gram)
3. .5g (half gram)
4. .75g (three quarters gram)
5. 1 g
6. 1.5g
7. 2.
8. 2.5g
9. 3g
10. 3.5g
11. 4g
12. 4.5g
13. 5g
14. Other. *Specify amount: _____*
15. In the past month, what was the **THC content** of the bud, weed, or other leaf products that you typically used?
    1. _____%
    2. Don’t know
    3. Prefer not to answer
16. In the past month, what was the **strain** of the cannabis that you typically used?
    1. __________
    2. Don’t know
    3. Prefer not to answer

Concentrated cannabis products (e.g. oils, vaping cartridges, resin, hash)

- - 1. How many **days** in the past month did you use concentrated cannabis products (e.g. oil, resin)?

____ *If 0, skip to Question 4*

Please refer to the image below depicting various quantities of marijuana. The image is not to scale; The bottle cap is included to help provide size perspective


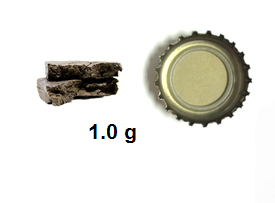


**Hash**


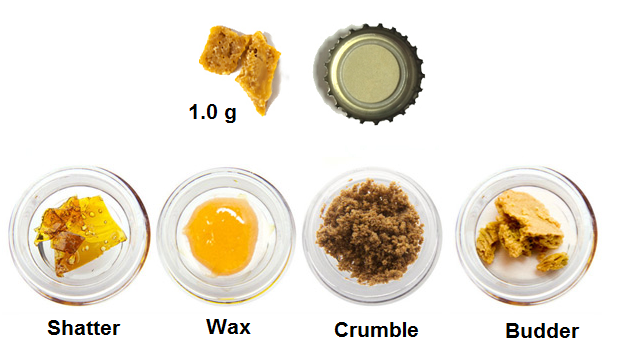


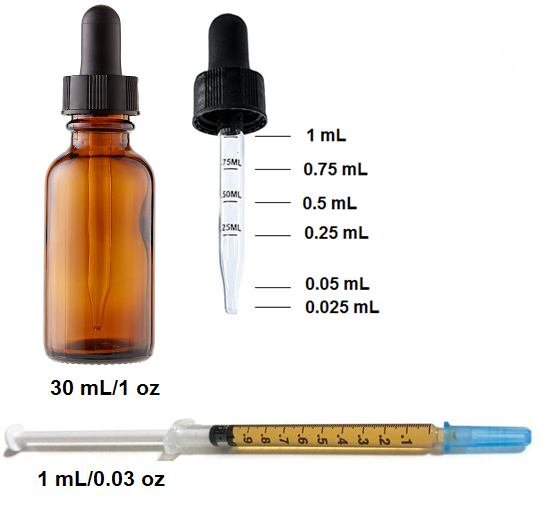


- - 1. How many **days** in the past month did you use the following types of concentrated cannabis products?
  1. Oil: __
  2. Resin: __
  3. Shatter: __
  4. Hash: __
  5. Other (please specify): _______ days: __
  6. NA - did not use any  *skip to question 5*
     1. On the days in the past month that you used concentrated products, how many **sessions** (blocks of time separated by at least 2-hours where you did not use cannabis) did you typically have in a **single day**?

1. 1
2. 2
3. 3
4. 4
5. 5
6. More than 5 (specify number): __
   - 1. In the past month, what is the **main way** that you used cannabis concentrates?
   1. Pipe
   2. Bong
   3. Joint
   4. Vaping/Shatter pen
   5. Ingestion
   6. Dabbing
   7. Hot knives
   8. Other: _____
   9. NA – I did not use this type
      1. In the past month, how many hits (1 hit = 1 puff, 1 drop) of concentrated cannabis products did you typically use **in a single day**?

_____ puff/drop/other:_______

12a. If you used drops, how much THC is in the product?

_____mg of THC per 1mL

- - 1. In the past month, how much **THC** was typically in the concentrated cannabis products that you used?
  1. _____%
  2. Don’t know
  3. Prefer not to answer

Edibles

- - 1. How many **days** in the past month did you consume edibles?

___ *If 0, skip to end of section*

- - 1. How many **days** in the past month did you consume the following types of edibles?
  1. Candy (e.g. gummies, mints): __
  2. Beverages (e.g. soda, teas, tinctures): __
  3. Chocolates: __
  4. Cookies or other baked goods: __
     1. On the days that you used edibles in the past month, how many **sessions** (blocks of time separated by at least 2-hours where you did not use cannabis) did you typically have **in a single day**?

1. 1
2. 2
3. 3
4. 4
5. 5
6. More than 5 (specify number): __
   - 1. In the past month, how much cannabis (in grams) did you typically consume through edibles **in a single day**?
7. ___ g
8. Don’t know
9. Prefer not to answer
   - 1. How many milligrams of THC did you typically ingest from edibles **in a single day**?
10. ____mg
11. Don’t know
12. Prefer not to answer
    - 1. In the past month, did you typically use store bought or homemade edible products?
13. Always store bought
14. Store bought most of the time (75% or more)
15. Store bought half the time
16. Homemade most of the time (75% or more)
17. Always homemade
    - 1. How many separate **days** in the past month did you use any cannabis product (0 to 31)?

____

**Part II – Other Factors Associated with Cannabis Use**

- - 1. Do you typically use cannabis alone or with other people?

1. Always or almost always alone
2. Alone half the time
3. Always or almost always with other people
4. Prefer not to answer
   - 1. How do you usually obtain your cannabis?
   1. Dealer
   2. Whatever friends have
   3. Home grown
   4. From a government-run cannabis store (online or in-person)
   5. From a non-regulated store (online or in person)
   6. Prefer not to answer
5. Do you typically use cannabis with alcohol or other substances?
   1. YES (specify substance): ________
   2. NO
   3. Prefer not to answer

**Part III – History**

1. How old were you when you first used cannabis?
2. How old were you when you started using it occasionally (i.e. once or twice a month)?
3. How many years altogether have you been using cannabis occasionally (i.e. once or twice a month)?
4. How old were you when you started using it regularly (i.e. at least weekly)?
5. How many years altogether have you been using cannabis regularly (i.e., at least weekly)?

1. Have you ever attempted to cut down or reduce your cannabis use?
   1. NO
   2. YES
2. Have you ever sought treatment from a physician, another health provider or counsellor, or informal support to reduce your cannabis use?
   1. NO
   2. Yes

Scoring: To calculate overall estimates of cannabis and THC used in the previous month, the following questions must be coded as numerical values:

Q4, Q5, Q6 (if provided), Q10, Q13 (if provided), Q16, Q17 (if provided), Q18 (if provided)

The following estimates can then be calculated:

Dry leaf

Sessions per day = Q4

Total sessions = Q2 * Q4

Amount per day =Q5

THC per day = (Q6/100) * Q5

Total cannabis = Q5 * Q2

Total THC = THC per day * Q2

Concentrates

Total sessions = Q8 * Q10

THC per day

*If reported hits in puffs*

THC per day (in grams) = ((Q13/100) * 5.2) / 1000

*if reported in drops (note: 1mL = 20 drops)*

THC per day (in grams) = ((Q12*0.025)*12a)/1000

Total THC = THC per day*Q8

Edibles

Total sessions = Q14 * Q16

Total cannabis = Q17*Q14

Total THC (in grams) = (Q18*Q14)/1000
